# Relationship between the dietary inflammatory index and immune function during pregnancy – A secondary analysis of the MicrobeMom2 Study

**DOI:** 10.1038/s41430-025-01690-2

**Published:** 2025-12-20

**Authors:** S. Hempelmann Perez, G. Mealy, K. Brennan, S. L. Killeen, R. Saldova, D. Groeger, D. Van Sinderen, P. D. Cotter, S. L. Doyle, F. M. McAuliffe

**Affiliations:** 1 UCD Perinatal Research Centre, School of Medicine, University College Dublin, National Maternity Hospital, Dublin, D02 YH21 Ireland; 2 Department of Clinical Medicine, Trinity College Institute of Neuroscience, School of Medicine, Trinity College Dublin, Dublin, 2 D02 PN40 Ireland; 3 NIBRT GlycoScience Group, National Institute for Bioprocessing Research and Training, Belfield, Blackrock, Co., Dublin, Ireland; 4 UCD School of Medicine, College of Health and Agricultural Science (CHAS), University College Dublin (UCD), Dublin, D04 R7R0 Ireland; 5 PrecisionBiotics Group Ltd (Novozymes), Cork Airport Business Park, Kinsale Road, Cork, T12 N84F Ireland; 6 Teagasc Food Research Centre, Moorepark, Fermoy, Cork, P61 C996 Ireland; 7 School of Microbiology, University College Cork, Cork, T12 K8AF Ireland; 8 APC Microbiome Ireland, University College Cork, Cork, T12 K8AF Ireland

**Keywords:** Nutrition, Health services

## Abstract

**Background/objectives:**

The maternal immune system requires careful regulation during pregnancy to prevent complications such as preeclampsia and prematurity. Inflammatory immune states may be linked to maternal diet throughout pregnancy. Although gestational changes in cytokines are well-documented, the association with the inflammatory potential of diet has not been robustly explored. This study investigates the associations between maternal dietary inflammatory potential, measured by energy adjusted dietary inflammatory index (E-DII) in late pregnancy (28-32 weeks’ gestation).

**Subjects/methods:**

E-DII was calculated using 6-month food frequency questionnaires completed by pregnant mothers at 28–32 weeks’ gestation. Serum cytokine levels from maternal blood samples were measured using Cobas® and ProteinSimple ELLA immunoassay, while PBMC derived cytokine levels were assessed using BioLegend LEGENDplex™.

**Results:**

The study included 68 pregnant women, with a mean age (SD) at recruitment of 33.01(4.6) years and median BMI (IQR) of 24.95 (21.87, 27.57) kg/m^2^. There was a positive association between E-DII and serum C3 complement protein in late pregnancy [β =0.057, *p* = 0.043]. A positive association between maternal E-DII and late pregnancy serum IL-17 levels was also observed [β = 0.156, *p* = 0.011]. A more pro-inflammatory diet in late pregnancy indicated by higher E-DII scores was associated with lower IL-10 secretion from PBMCs stimulated with anti-CD3/28/2 [β = −0.232, *p* = 0.049].

**Conclusions:**

Diet may hold potential to promote optimal maternal inflammatory states. These data may inform nutritional guidelines to reduce pregnancy related complications, especially for mothers with higher background metabolic risk, such as those living with obesity.

## Introduction

A mother’s immune system undergoes significant and complex changes throughout pregnancy to protect the fetus [1, 2]. It has been suggested that the first and third trimesters are characterised by a generally pro-inflammatory state to support implantation and delivery, respectively, while the second trimester is anti-inflammatory to allow for fetal development [3–5]. Implantation and delivery are generally pro-inflammatory to support blastocyst attachment and tissue remodeling, while the second trimester is anti-inflammatory to promote fetal growth and immune tolerance [5, 6]. These processes must be tightly regulated as excessive inflammatory states have been linked with increased rates of preeclampsia, premature birth, and adverse long-term health outcomes for newborns [7]. Furthermore, women who have a higher Body Mass Index (BMI) may be at higher risk of maternal and child inflammatory-related health disorders, as evidence suggests they may have increased baseline inflammatory states marked by elevated levels of C3 or C-reactive protein (CRP) [8–10].

While some cytokines, such as IL-10 have been shown to positively affect pregnancy through maternal immune tolerance of the fetus, other pro-inflammatory cytokines such as IL-2, TNF-α and IFN-γ have demonstrated a negative impact on pregnancy success when their expression is dysregulated [11–14]. In the non-pregnant population, healthier diets, evidenced by a higher consumption of fruits and vegetables, are associated with lower levels of systemic inflammation, demonstrated by decreased plasma levels of CRP [15].

The dietary inflammatory index (DII), designed by Shivappa et al., is a literature-derived tool that categorises dietary patterns according to their inflammatory potential [16]. The energy adjusted dietary inflammatory index (E-DII) is more robust, as it adjusts for energy intake, which can largely vary across individuals [17]. Mothers with a higher intake of fruit, vegetables, whole grains, and nuts have lower dietary inflammatory indexes, whereas those with a higher consumption of fatty, processed, and fast foods have higher E-DIIs [18]. It is possible that diet plays a role in modulating the immune response in pregnancy. One recent study showed that E-DII scores during early pregnancy were positively associated with IL-6 levels [19]. Other studies have shown that a mother’s E-DII during pregnancy is related to maternal cardiometabolic health, neonatal adiposity, lower offspring birth size and affects perinatal asphyxia [18–22]. Maternal diet is an important modifiable factor during pregnancy, especially for mothers who may be at increased risk of adverse pregnancy outcomes [23]. Considering the potential impact of the inflammatory component of a mother’s diet on offspring’s health, further studies are needed to elucidate the interaction between maternal E-DII and the immune system.

Few studies have investigated the association between the dietary inflammatory index and a wide range of serum cytokine levels during pregnancy. Reports have primarily been limited to IL-6, IL-8, TNF-α and CRP. Furthermore, the connection between the E-DII and anti-inflammatory cytokines is sparsely reported. Similarly, there is a paucity of data analysing the associations between E-DII and Peripheral Blood Mononuclear Cell (PBMC) derived cytokine levels, which our study addresses. Investigating more serum and PBMC inflammatory and anti-inflammatory biomarkers may help to better understand the complexities of the relationship between the E-DII and the immune response during pregnancy and help to address discrepancies in the literature [24].

This study aims to examine the correlations between E-DII scores in pregnant mothers and serum levels of CRP, C3, ICAM-1, TNF-α, GDF-15, sCD163, leptin, IL-6 and IL-17, and PBMC derived cytokine levels, of lL-2, IL-10, IL-6, IFN-γ & TNF-α.

## Methods

### Study design & participants

The MicrobeMom2 Study, conducted at the National Maternity Hospital of Ireland between 2020 and 2022, was a double-blind randomised control trial (RCT) investigating the impact of probiotic supplementation on the maternal cytokine response [25]. This double-blinded randomised-controlled trial involved oral supplementation of *Bifidobacterium longum subsp. longum* 1714® (*B. longum* 1714; daily ingestion of a minimum of 1×10^9^ colony forming units) or placebo from 16 to 20-weeks’ gestation until delivery in healthy pregnant women. The primary outcome was a change in IL-10 production, after stimulation with Lipopolysaccharide (LPS) or anti-CD3/28/2, in PBMCs isolated from blood samples taken at baseline (11–15 weeks’ gestation) and late pregnancy (28–32 weeks’ gestation) after 48 h incubation.

This study is a secondary analysis of 68 pregnant women from the Microbemom2 RCT and reports new analyses investigating the associations between E-DII and inflammatory biomarkers. As this was a secondary analysis, it was not powered; however, details of sample size calculation for the primary outcome paper are shown elsewhere [25]. The inclusion criteria were mothers aged 18 to 45 years, ability to understand English, early pregnancy BMI of 18.5–35 kg/m^2^, and ability to give informed consent. Mothers who met the inclusion criteria following the first antenatal visit at 10–15 week’s gestation were approached for participation in the study. Participants with a multiple pregnancy, previous miscarriage, fetal anomaly, history of gestational diabetes (GDM), diabetes mellitus or pre-diabetes, other medical conditions requiring treatment or unwillingness to adhere to the trial’s supplement or probiotic regime were excluded from the study. All participants gave written, informed consent prior to recruitment. No specific diet was recommended during the study, except to refrain from foods or supplements high in probiotics.

### Data collection

Demographic information for all mothers was collected at the initial visit (11–15 weeks gestation) and included maternal age, ethnicity, parity and current smoking status. Maternal weight (to the nearest 0.1 kg) using the SECA weighting scale (SECA GmbH & co. kg., Hamburg, Germany) and maternal height (to the nearest 0.1 cm) using a stadiometer were measured. Values for maternal weight and height were collected wearing light clothing and without footwear, respectively. These measurements were then used to calculate BMI.

#### Dietary analysis

An adapted version of the Food4Me Food Frequency Questionnaire (FFQ) was completed by mothers during late pregnancy and was used to measure the mother’s dietary intake during the past 6 months [26]. FFQs are one of the most common methods used to measure dietary and nutritional food intake, and have been validated in pregnant women [27]. For each question, mothers were asked their average intake of 200 foods, on a Likert scale of “Never or less than once per month” to “6+ times per day.” Food categories included: meat/fish/poultry, carbohydrates, dairy products and fats, fruit, vegetables, sweets and snacks, soups/sauces/spreads, and drinks.

This dietary data was used for computation of the E-DII. The E-DII is based on 45 food parameters which have been demonstrated in the literature to be associated with six inflammatory biomarkers in non-pregnant individuals: IL-1β, IL-4, IL-6, IL-10, TNF-α and CRP [16]. This index has since been validated for use in the pregnant population as well [19, 28]. In our study, 28 of the 45 food parameters were available from the FFQ and were used to calculate an individual’s overall E-DII score. The 28 food parameters included: Energy, Carbohydrate, Protein, Fat, Alcohol, Fiber, Cholesterol, SFA, MUFA, PUFA, Omega 3, Omega 6, Trans Fat, Niacin, Thiamin, Riboflavin, B12, B6, Iron, Magnesium, Zinc, Selenium, Vitamin A, Vitamin C, Vitamin D, Vitamin E, Folic Acid and Caffeine. Exact methods for calculating the E-DII are described in previous studies which used the index [16, 17]. Briefly, a z-score was derived for the 28 nutrients by subtracting the global mean from the participant’s nutrient intake value and dividing by the global standard deviation. Z-scores were then converted to proportions and centered by doubling each score and subtracting one. Next, scores were multiplied by their global inflammatory effect scores. Finally, all 28 scores were summed to obtain the overall E-DII score and reported per 1000 kcal consumption. A negative score suggests an anti-inflammatory diet while a positive scores suggests a pro-inflammatory diet [16].

Since women, including pregnant women, have shown to underreport dietary intakes, this must be accounted for [29, 30]. To estimate the extent of dietary underreporting, Goldberg’s method was used, by dividing energy intake by estimated basal metabolic rate [31]. To calculate basal metabolic rate, Schofield’s equation including mother’s weight (kg) and age (years) was employed [32]. Definite under-reporters were described as those with a ratio of <0.9 [31]. Dietary underreporting was included as a covariate in the linear regression models to account for its potential influence on the results.

#### PBMC

Blood samples of all mothers were taken during late pregnancy, at 28–32 weeks’ gestation.

Following the collection of maternal venous blood samples in K2EDTA tubes, PBMCs were cryopreserved using 90% fetal bovine serum (FBS) and 10% dimethyl sulfoxide (DMSO) (Stemcell). PBMCs were then isolated by density gradient centrifugation using LymphoprepTM (Stemcell). Detailed methodology of PBMC isolation and storage are described in the Microbemom2 study [25]. To stimulate PBMCs, one of two stimulants were used to elicit an immune response: Lipopolysaccharide (LPS, (24 h, 100 ng/ml) (Enzo)) to activate the Toll-like receptor 4 (TLR4), or CD3/CD28/CD2 (CD3/28/2, 48 h (Stemcell)), to activate T cells. Following stimulation, a BioLegend LEGENDplex™ Human Inflammation Panel (5-plex) was carried out as per manufacturer’s instructions to determine levels of IL-10, TNFα, IFNγ, IL-6 and IL-2 in PBMC supernatants. A BD LSR Fortessa cell analyser was used to acquire samples and BioLegend LEGENDplex™ software was used for analysis.

#### Serum

Maternal serum samples were also collected in late pregnancy and stored in VACUETTE serum tubes (Greiner Bio-One, Austria) at -80 °C. To isolate serum from whole blood, centrifugation was done using the Rotina 420 R (Hettich, MA, USA) for 10 min at 3000 rpm with the break and accelerator on setting 3. The Cobas® system (Roche, Basil) was used to determine serum concentrations of CRP and C3. The ProteinSimple ELLA automated immunoassay system (Biotechne, Minneapolis) was used to determine the levels of IL- 17 A, IL-6, TNF-α, CD163, ICAM1, GDF-15, and Leptin. The cytokines selected aim to offer further understanding regarding aspects of the immune and metabolic responses during pregnancy. These include inflammatory responses, demonstrated by IL-17A, IL-6, and TNF-α; anti-inflammatory responses, shown through CD163 and GDF-15; leukocyte adhesion, indicated by ICAM1; and metabolic processes, observed through GDF-15 and Leptin.

### Statistical analysis

IBM SPSS version 27.0 for Windows (SPSS Inc, Chicago, IL) was used to run all statistical analyses. Participant demographics were generated by using frequency distributions and descriptive statistics. Assessment of normality was done by inspection of histograms, Shapiro-Wilk tests, and descriptive data such as mean, median, kurtosis, and skewness. Data that was skewed was log^10^ transformed, and normality of transformed data was assessed before undergoing further analysis. Linear regressions were run to examine the associations between serum cytokine levels and E-DII in late pregnancy. A secondary linear regression was also conducted to assess the link between PBMC-derived cytokines and E-DII in late pregnancy.

Covariates were included based on a review of factors known to affect the maternal immune response [33–35]. Maternal ethnicity was included as previous studies have demonstrated variations in baseline immunological functioning amongst different ethnic groups of pregnant women, which could potentially affect both serum and PBMC derived cytokine levels [36, 37]. Smoking was also included as a covariate due to the increased pro-inflammatory states that have been reported with maternal smoke exposure [34]. Final analysis models adjusted for: maternal parity (nulliparous, multiparous), maternal smoking status (yes, no), maternal ethnicity (white Irish, other), original study group (probiotic, placebo) and maternal early pregnancy BMI (healthy, overweight, obese). World Health Organization guidelines were used to classify participants into healthy (18.5–24.9 kg/m^2^), overweight (25–29.9 kg/m^2^) and obese (30–35 kg/m^2^) categories. Another model was also run which adjusted for all the covariates above, as well as dietary underreporting (dietary under-reporter, not). Unadjusted linear regression results were also reported. Alpha levels of *p* < 0.05 were used to assess statistical significance.

## Results

### The microbemom2 RCT

The primary study, a randomised controlled trial of probiotic supplementation with Bifidobacterium longum 1714® versus placebo for a 12-week period during pregnancy, found no significant differences in cytokine production between intervention and control groups in either PBMC or serum cytokine markers. Further detail is available in the primary study publication [25].

### Demographics

Maternal demographics are presented in Table 1. The primary analysis of the MicrobeMom2 Study included 72 women. For this secondary analysis, four participants were excluded due to missing data: two lacked smoking status information, and two had incomplete food frequency questionnaire data. The final sample therefore comprised 68 women. There were no cases of preterm birth, neonatal death, gestational hypertension, pre-eclampsia or congenital anomalies [25]. Mean E-DII score was −0.45 (1.13), with a range of -3.06 to 2.46. Median pregnancy-based BMI (IQR) was 24.95 (21.87, 27.57) kg/m^2^. Mean age (SD) of mothers was 33.06 (4.60), and the majority (82.4%) were Irish. 14 (20.6%) of women were defined as dietary under reporters according to Goldberg’s method [31] and were adjusted for in one of our models. Baseline serum cytokine concentrations and PBMC biomarker fold-changes in late pregnancy are included in Table 2.

**Table 1:** (*n* = 68) Maternal characteristics.

	Value
Age (years)^a^	33.1 (4.6)
Body Mass Index (kg/m^2^)^b^	24.95 (21.87, 27.57)^b^
- Healthy (18-24.9), n (%)	37 (54.4%)
- Overweight (25-29.9), n (%)	22 (32.4%)
- Obesity (30-35), n (%)	9 (13.2%)
Ethnicity, n (% White Irish)	56 (82.3%)
- Other white background	8 (11.8%)
- Other mixed background	2 (2.9%)
- Black or African American	1 (1.5%)
- Other	1 (1.5%)
Smoking in Pregnancy, n (%)	2 (2.9%)
Nulliparous, n (%)	20 (29.4%)
Dietary under reporters, n (%)*	14 (20.6%)
E-DII^a^	−0.45 (1.13)
Study Group, n (% Probiotic Group)	35 (51.5%)
Maternal Nutrient Intakes	
- Energy (kcal)^a^	1862.16 (706.86)
- Carbohydrate (g)^a^	231.22 (96.82)
- Protein (g)^a2314^	84.03 (34.31)
- Fat (g)^a^	72.14 (27.79)
- Alcohol (g)^a^	1.60 (3.92)
- Fiber (g)^a^	24.35 (10.36)
- Cholesterol (mg)^a^	329.00 (171.15)
- SFA (g)^a^	29.32 (11.72)
- MUFA (g)^a^	26.42 (10.11)
- PUFA (g)^a^	12.04 (5.20)
- Omega 3 (g)^a^	1.58 (1.71)
- Omega 6 (g)^a^	3.86 (1.67)
- Trans Fat (g)^a^	1.27 (0.59)
- Niacin (mg)^a^	24.96 (11.08)
- Thiamin (mg)^a^	2.05 (0.79)
- Riboflavin (mg)^a^	1.61 (0.68)
- Vitamin B12 (μg)^a^	4.70 (2.28)
- Vitamin B6 (mg)^a^	1.88 (0.70)
- Iron (mg)^a^	11.55 (4.72)
- Magnesium (mg)^a^	303.48 (122.61)
- Zinc (mg)^a^	9.51 (3.80)
- Selenium (μg)^a^	53.90 (23.45)
- Vitamin A (RE)^a^	373.91 (194.29)
- Vitamin C (mg)^a^	154.10 (88.16)
- Vitamin D (μg)^a^	3.01 (1.61)
- Vitamin E (mg)^a^	10.96 (3.98)
- Folic Acid (μg)^a^	334.38 (140.75)
- Caffeine (g)^a^	0.08 (0.06)

Data is presented as mean (standard deviation (SD))^a^ for parametric values or median (interquartile range 25th, 75th)^b^. The n column marks the number of participants with available data. The secondary n (%) represents the participants corresponding to the demographic indicated by the row. *Indicates definite dietary under-reporters. Definite under-reporters were defined as mothers having a Goldberg ratio of < 0.9. Percentages are given to one decimal place and Mean (SD) and median (IQR) are given to two decimal places.

**Table 2:** Serum and PBMC Biomarkers for Late Pregnancy.

	Sample size	Stimulation *stimulant (incubation time)*	Late Pregnancy *median (IQR)*
**Serum**			
CRP (mg/L)	n = 68	–	3.33 [2.12, 5.31]
C3 (g/L)	n = 68	–	1.83 [1.70, 2.03]
ICAM-1 (ng/mL)	n = 67	–	300.49 [253.14, 352.26]
TNF-α (pg/mL)	n = 67	–	8.44 [7.43, 9.63]
GDF-15 (ng/mL)	n = 67	–	15.18 [12.62, 18.19]
sCD163 (ng/mL)	n = 67	–	493.82 [390.90, 593.00]
Leptin (ng/mL)	n = 67	–	29.54 [16.71, 42.16]
IL-6 (pg/mL)	n = 67	–	1.58 [1.22, 2.00]
IL-17A (pg/mL)	n = 67	–	0.81 [0.44, 1.14]
**PBMC**			
IL-2 (fold change)	n = 68	Anti-CD3/28/2 (48 h)	6003.65 [1071.50, 43017.33]
IL-10 (fold change)	n = 68	LPS (24 h)	34.77 [6.54, 395.52]
IL-6 (fold change)	n = 68	LPS (24 h)	69.76 [9.48, 812.37]
IFN-y (fold change)	n = 68	Anti-CD3/28/2 (48 h)	17057.81 [864.06, 196135.50]
TNF-α (fold change)	n = 68	LPS (24 h)	250.44 [8.59, 2506.68]

Serum and PBMC immune markers at late pregnancy (28–32 weeks’ gestation). PBMCs were stimulated with either LPS for 24 h or Anti-CD3/28/2 (48 h).

### Impact of E-DII on serum cytokine levels

Table 3 describes the linear regression models analysing the associations between E-DII and serum cytokine levels in late pregnancy. In late pregnancy, a higher E-DII was positively associated with levels of C3 in Model 1 (ß = 0.066, *p* = 0.022), Model 2 (ß = 0.056, *p* = 0.047) and Model 3 (ß = 0.057, *p* = 0.043). In late pregnancy, E-DII was significantly positively associated with serum IL-17A levels (ß = 0.162, *p* = 0.005), which remained significant following adjustment with Model 2 covariates (ß = 0.154, *p* = 0.012) and Model 3 covariates (ß = 0.156, *p* = 0.011). No significant differences were found between E-DII and serum cytokine levels of CRP, ICAM-1, TNF-α, GDF-15, sCD163, leptin and IL-6 in late pregnancy for all models. Analyses were also controlled for age although no difference was noted for all outcomes and models (Data not shown).

**Table 3:** Associations between E-DII score and serum markers of inflammation.

	Late Pregnancy		
Serum	ß (95% CI)	*P*-value	Adj. R^2^
**CRP** (mg/L)	*n* = 68		
Model 1^a^	0.038 [−0.029, 0.105]	0.265	0.004
Model 2^a, b^	0.047 [−0.023, 0.117]	0.186	−0.003
Model 3^a, c^	0.049 [−0.022, 0.119]	0.173	−0.011
**C3** (g/L)	*n* = 68		
Model 1	0.066 [0.010, 0.122]	0.022*	0.063
Model 2^b^	0.056 [0.001, 0.111]	0.047*	0.159
Model 3^c^	0.057 [0.002, 0.113]	0.043*	0.155
**ICAM-1** (ng/mL)	*n* = 68		
Model 1^a^	−0.005 [−0.030, 0.020]	0.710	−0.013
Model 2^a, b^	−0.002 [−0.028, 0.024]	0.885	−0.025
Model 3^a, c^	−0.002 [−0.029, 0.024]	0.851	−0.034
**TNF-α** (pg/mL)	*n* = 68		
Model 1	0.000 [−0.499, 0.498]	0.999	−0.015
Model 2^b^	−0.011 [−0.528, 0.507]	0.967	−0.013
Model 3^c^	−0.012 [−0.534, 0.511]	0.965	−0.030
**GDF-15** (ng/mL)	*n* = 68		
Model 1^a^	−0.003 [−0.041, 0.035]	0.880	−0.015
Model 2^a, b^	0.005 [−0.035, 0.045]	0.809	−0.046
Model 3^a, c^	0.004 [−0.036, 0.044]	0.838	−0.059
**sCD163** (ng/mL)	*n* = 68		
Model 1^a^	−0.007 [−0.040, 0.027]	0.690	−0.013
Model 2^a, b^	−0.004 [−0.038, 0.029]	0.792	0.036
Model 3^a, c^	−0.003 [−0.036, 0.031]	0.872	0.059
**Leptin** (ng/mL)	*n* = 68		
Model 1^a^	0.006 [−0.054, 0.067]	0.838	−0.015
Model 2^a, c^	0.010 [−0.041, 0.062]	0.695	0.315
Model 3^a. c^	0.011 [−0.040, 0.063]	0.659	0.311
**IL-6** (pg/mL)	*n* = 68		
Model 1^a^	0.002 [−0.039, 0.044]	0.910	−0.015
Model 2^a, b^	0.005 [−0.036, 0.047]	0.799	0.061
Model 3 ^a, c^	0.005 [−0.037, 0.047]	0.814	0.046
**IL-17A** (pg/mL)	*n* = 68		
Model 1	0.162 [0.050, 0.275]	0.005*	0.098
Model 2^b^	0.154 [0.034, 0.273]	0.012*	0.064
Model 3^c^	0.156 [0.037, 0.276]	0.011*	0.057

Values determined using linear regression models; all results presented as unstandardised beta coefficient (95% confidence interval). *Statistically significant (*p*-value < 0.05). *E-DII* Energy adjusted Dietary Inflammatory Index, *B* Beta coefficient, *CI* Confidence interval, *Adj* Adjusted, *BMI* Body mass index, *CRP* C-reactive protein, *ICAM-1* Intracellular adhesion molecule-1, *TNF-α* Tumor necrosis factor-alpha, *GDF-15* Growth of differentiation factor-15, *sCD163* Soluble cluster of differentiation factor-163, *IL* Interleukin. ^a^Log10 transformed data was used. Model 1 is unadjusted. ^b^Model 2 is adjusted for maternal parity, maternal smoking status, maternal early pregnancy BMI, maternal ethnicity, original study group. ^c^Model 3 is adjusted for maternal parity, maternal smoking status, maternal early pregnancy BMI, maternal ethnicity, original study group and dietary underreporting.

### Impact of E-DII on PBMC-derived markers of inflammation

Table 4 illustrates the linear regression models that examine the associations between E-DII and PBMC-derived cytokine levels in late pregnancy. This table shows that without adjusting for confounders, maternal E-DII was significantly negatively associated with IL-10 levels in late pregnancy. Following adjustment with Model 2 confounders, including maternal parity, maternal smoking status, maternal early pregnancy BMI, maternal ethnicity and original study group, the association between higher maternal E-DII score in late pregnancy and lower levels of IL10 was not observed. In contrast, when adjusting for the aforementioned confounders and dietary underreporting (Model 3), a higher maternal E-DII was associated with lower levels of IL-10. We observed no significant association between maternal E-DII and PBMC-derived cytokine levels of IL-2, IL-6, IFN-γ and TNF-α in late pregnancy. Regression analyses between DII and ratios of PBMC-derived IL10/TNF-α and IL10/IFN-γ were non-significant across all models and were therefore not included.

**Table 4:** Associations between E-DII score and PBMC-derived markers of inflammation.

	Late Pregnancy		
PBMC	ß (95% CI)	*P*-value	Adj. R^2^
**IL-2**^a^	*n* = 68		
Model 1^b^	−0.028 [−0.281, 0.224]	0.825	−0.014
Model 2^b, c^	−0.044 [−0.316, 0.229]	0.750	−0.094
Model 3^b, d^	−0.037 [−0.311, 0.237]	0.789	−0.102
**IL-10**^e^	*n* = 68		
Model 1^b^	−0.267 [−0.490, −0.044]	0.020*	0.066
Model 2^b, c^	−0.220 [−0.452, 0.012]	0.063	0.061
Model 3^b, d^	−0.232 [−0.462, −0.001]	0.049*	0.080
**IL-6**^e^	*n* = 68		
Model 1^b^	0.055 [−0.200, 0.310]	0.668	−0.012
Model 2^b, c^	0.047 [−0.218, 0.312]	0.723	−0.014
Model 3^b, d^	0.039 [−0.227, 0.304]	0.773	−0.015
**IFN-γ**^a^	*n* = 68		
Model 1^b^	−0.117 [−0.471, 0.237]	0.511	−0.008
Model 2^b, c^	−0.084 [−0.463, 0.296]	0.661	−0.075
Model 3^b, d^	−0.092 [−0.474, 0.290]	0.632	−0.085
**TNF-α**^e^	*n* = 68		
Model 1^b^	−0.168 [−0.494, 0.158]	0.306	0.001
Model 2^b, c^	−0.120 [−0.461, 0.222]	0.487	−0.019
Model 3^b, d^	−0.140 [−0.477, 0.196]	0.407	0.019

Values determined using linear regression models; all results presented as unstandardised beta coefficient (95% confidence interval). *Statistically significant (*p*-value < 0.05). *E-DII* Energy adjusted Dietary Inflammatory Index, *B* Beta coefficient, *CI* Confidence interval, *Adj* Adjusted, *BMI* Body mass index, *IL* Interleukin, *TNF-α* Tumor necrosis factor-alpha, *IFN- γ* Interferon gamma. ^a^PBMCs stimulated with Anti-CD3/28/2 for 48 h. ^b^Log10 transformed data was used. Model 1 is unadjusted. ^c^Model 2 is adjusted for maternal parity, maternal smoking status, maternal early pregnancy BMI, maternal ethnicity, original study group. ^d^Model 3 is adjusted for maternal parity, maternal smoking status, maternal early pregnancy BMI, maternal ethnicity, original study group and dietary underreporting. ^e^PBMCs stimulated with LPS for 24 h.

## Discussion

### Main findings

Our study sought to investigate the associations between dietary inflammatory potential during pregnancy and serum and PBMC-derived cytokines. There was no relationship between E-DII and levels of CRP, ICAM-1, TNF-α, GDF-15, sCD163, Leptin, IL-6, IL-2 and IFN-γ; however, our study yields new data on the relationship between E-DII and serum levels of C3, IL-17A and PBMC-derived IL-10. In our cohort, a higher E-DII was positively associated with serum C3 complement component (C3) in late pregnancy. In late pregnancy, serum levels of IL-17A were significantly higher in mothers consuming a more pro-inflammatory diet. We also observed that a more pro-inflammatory diet at this timepoint was associated with decreased levels of PBMC-derived IL-10 in two of our models.

The complement system, including C3, is involved in bridging the innate and adaptive immune response. C3 is primarily produced by the liver but can also be synthesised by the placenta during pregnancy [38]. Our findings for C3 are similar to those found by Killeen et al., where a relationship between E-DII and concentrations of C3 were observed amongst pregnant women with overweight and obesity, although significance in that study was lost following adjusting for BMI and other confounders [39]. The association between a less inflammatory diet and decreased C3 has been reported in non-pregnant cohorts [40]. Past studies have recognised C3 as a significant risk factor for cardiometabolic disease [41]. A significant association between maternal C3 and markers of maternal insulin resistance, altered lipid metabolism and gestational diabetes has been reported [9]. Furthermore, higher levels of C3 have been shown to have a strong relationship with pre-term delivery and fetal loss [42, 43]. Therefore, increased levels of C3 due to a more inflammatory diet may be a potential modifiable risk factor for mothers.

IL-17A is an inflammatory cytokine that is primarily produced by T helper 17 cells (Th17) [44]. The protein is widely involved in promoting inflammation, as IL-17A receptors are ubiquitously expressed throughout the body [45]. Only one other study to date has examined the association between dietary inflammation and levels of IL-17A in pregnant cohorts, and no association was found [46]. However, this may be due to the fact that cytokine levels were measured in early pregnancy (around 12 weeks). Numerous studies have shown that IL-17A plays a crucial role in driving obesity-related inflammation, with Th17 cells found to be increased in adipose tissues of obese animals and humans [45, 47]. The impact of IL-17A related inflammation may also extend beyond its role in obesity. Another study by Moore et al., found that increased levels of IL-17A during late pregnancy were associated with higher prenatal distress and lower levels of maternal attachment [48]. Our findings are important since regulating IL-17A levels through maternal diet may have a positive preventative impact on the development of chronic inflammatory diseases and even psychosocial distress [44].

IL-10 has been extensively studied in E-DII research and pregnancy due to its established role in promoting positive pregnancy outcomes [49]. It was also one of the six cytokines that was used to develop the D-II [16]. It is an anti-inflammatory cytokine that exerts its effect by decreasing levels of pro-inflammatory cytokines, including IL-1, IL-6, IL-12 and TNF- α [13]. Our findings for IL-10 are consistent with another study by Pieczyńska et al. that showed a negative association between D-II and IL-10 in normal pregnancy, but only during the second trimester [50]. However, this may be due to the smaller sample size in their study, and that IL-10 levels were collected from serum rather than PBMCs. A similar study of pregnant mothers found a negative association between D-II and serum IL-10 in the third trimester [51]. These findings demonstrate that an inflammatory diet may result in a decrease in maternal anti-inflammatory cytokine concentrations such as IL-10. Deficiencies in IL-10 have been associated with recurrent spontaneous abortion, preterm birth, and pre-eclampsia, emphasising the importance of regulating IL-10 levels [13, 52].

### Clinical implications

Our study helps to increase the understanding of interplay between the inflammatory potential of maternal diet and the immune response. Dietary modifications hold potential to reduce pregnancy related inflammation and decrease the risk of pro-inflammatory related disorders such as hypertensive disorders of pregnancy and gestational diabetes. Our findings may also contribute to the evolution of nutritional guidelines during pregnancy, especially for mothers with increased inflammatory states such as those living with obesity and those with insulin resistance.

### Strengths, limitations & future research

A strength of this study is the novel use of PBMC-derived cytokine levels, as previous studies have primarily analysed serum cytokines. Another advantage is that the study was undertaken prospectively. We also used the E-DII, rather than the D-II, which adjusts for energy intake and is a more accurate measurement of dietary inflammatory potential [17, 22]. Further, while there is a sparsity of studies investigating the association between dietary inflammatory potential and the pro-inflammatory immune response, studies investigating D-II and anti-inflammatory cytokines are scarce.

A limitation of our study is that cytokine levels are reported for only one time point, which may not reflect the full maternal cytokine profile during pregnancy. Additionally, our sample was small and not ethnically diverse, which may limit the global applicability of our findings. Since our sample consisted of mothers who were generally healthy, our findings may also differ for women with adverse health conditions or pregnancy complications.

Our findings suggest that diet may contribute to maternal inflammation. Further studies replicating these findings and examining the impact of dietary modifications are needed, especially due to the long-term effects these changes may have on an offspring’s health. We also acknowledge that our study did not include mothers with higher BMIs (>35). Given the impact of obesity on the immune response during pregnancy, future studies including this population may yield important insights into inflammatory biomarker levels, such as IL-17A, C3 and CRP, and warrants further exploration [9, 53]. Finally, given the changes that occur in the immune response throughout pregnancy, future studies including multiple timepoints could be beneficial.

## Conclusion

Maternal nutrition during pregnancy plays a significant role in the short and long-term health of a mother and her children. Our study found that several inflammatory modulators, notably C3, IL-17A and IL-10 were significantly associated with E-DII. These data may inform nutritional guidelines to reduce pregnancy related complications, especially for mothers with higher background metabolic risk, such as those who are insulin resistant and those living with obesity.

## Data Availability

Data available upon request.
